# The Polyphenol Pterostilbene Ameliorates the Myopathic Phenotype of Collagen VI Deficient Mice via Autophagy Induction

**DOI:** 10.3389/fcell.2020.580933

**Published:** 2020-09-29

**Authors:** Samuele Metti, Lisa Gambarotto, Martina Chrisam, Martina La Spina, Martina Baraldo, Paola Braghetta, Bert Blaauw, Paolo Bonaldo

**Affiliations:** ^1^Department of Molecular Medicine, University of Padova, Padua, Italy; ^2^Department of Biomedical Sciences, University of Padova, Padua, Italy; ^3^Venetian Institute of Molecular Medicine, Padua, Italy; ^4^CRIBI Biotechnology Center, University of Padova, Padua, Italy

**Keywords:** skeletal muscle, autophagy, muscle remodeling, congenital muscular dystrophies, nutraceutical agent, Collagen VI

## Abstract

The induction of autophagy, the catabolic pathway by which damaged or unnecessary cellular components are subjected to lysosome-mediated degradation and recycling, is impaired in Collagen VI (COL6) null mice and COL6-related myopathies. This autophagic impairment causes an accumulation of dysfunctional mitochondria, which in turn leads to myofiber degeneration. Our previous work showed that reactivation of autophagy in COL6-related myopathies is beneficial for muscle structure and function both in the animal model and in patients. Here we show that pterostilbene (Pt)—a non-toxic polyphenol, chemically similar to resveratrol but with a higher bioavailability and metabolic stability—strongly promotes *in vivo* autophagic flux in the skeletal muscle of both wild-type and COL6 null mice. Reactivation of autophagy in COL6-deficient muscles was also paralleled by several beneficial effects, including significantly decreased incidence of spontaneous apoptosis, recovery of ultrastructural defects and muscle remodeling. These findings point at Pt as an effective autophagy-inducing nutraceutical for skeletal muscle with great potential in counteracting the major pathogenic hallmarks of COL6-related myopathies, a valuable feature that may be also beneficial in other muscle pathologies characterized by defective regulation of the autophagic machinery.

## Introduction

Macroautophagy (hereafter autophagy) is an evolutionarily conserved and multistep self-eating mechanism, by which damaged or unnecessary intracellular components are engulfed into specific double-membrane vesicles, called autophagosomes, and subjected to lysosome-mediated degradation and recycling (Mizushima, 2007). Autophagy is a very dynamic process that needs to be finely tuned in the different tissues and cell types. Indeed, any perturbation in autophagy regulation may seriously endanger cell viability and function, especially in post-mitotic cells (Levine and Kroemer, 2008).

Skeletal muscle is a highly plastic tissue, able to adapt its metabolism, mass and strength to several physiological conditions, via balancing protein synthesis and degradation (Schiaffino et al., 2013). In particular, the autophagic pathway plays a crucial role during muscle development and maintains cellular homeostasis and energy balance in mature fibers (Bonaldo and Sandri, 2013). Autophagy dysregulation is involved in the etiopathogenesis of various muscle disorders, such as muscular dystrophies, congenital myopathies, cachexia and sarcopenia (Grumati and Bonaldo, 2012; Castets et al., 2016). A well-characterized model of impaired autophagy induction in skeletal muscle is the COL6 null (*Col6a1^–/–^*) mouse (Bonaldo et al., 1998; Cescon et al., 2015), in which accumulation of dysfunctional mitochondria and abnormal organelles in myofibers leads to muscle wasting and weakness (Irwin et al., 2003; Grumati et al., 2010). Similar alterations are present in patients affected by Bethlem myopathy (BM) and Ullrich congenital muscular dystrophy (UCMD), two rare inherited muscle disorders caused by mutations of COL6 genes and for which no cure is yet available (Grumati et al., 2010; Bönnemann, 2011). Of note, defective regulation of autophagy was also reported in the dystrophin-deficient *mdx* mouse (De Palma et al., 2012; Spitali et al., 2013) and in the MTM1 null mouse (Fetalvero et al., 2013), animal models for Duchenne muscular dystrophy and X-linked myotubular myopathy, respectively.

In the last decade, targeted approaches aimed at promoting the autophagic flux gained increasing interest as a novel option for prospective therapeutic strategies in a range of disorders and pathological conditions (Rubinsztein et al., 2012). The easiest and most characterized way to boost autophagy is represented by caloric or nutritional restriction, which can be elicited by different means, such as fasting or decreased protein intake (Rubinsztein et al., 2012; Bagherniya et al., 2018). Regarding COL6-related myopathies, these approaches were shown to be beneficial both in preclinical studies in *Col6a1^–/–^* mice (Grumati et al., 2010; Chrisam et al., 2015) and in a pilot clinical trial in UCMD and BM patients, in which autophagy was successfully promoted through a 1-year-long low protein diet (Castagnaro et al., 2016). However, such dietary regimen unavoidably requires a deep and rigorous lifestyle change that may challenge patients’ compliance and the bench-to-bedside process. An increasingly attractive option for autophagy stimulation is represented by a broad range of nutraceutical compounds that act as caloric restriction mimetics (Madeo et al., 2019; Maiuri and Kroemer, 2019), such as the polyamine spermidine (Madeo et al., 2018).

Pterostilbene (trans-3,5-dimethoxy-4′-hydroxystilbene, Pt) is a non-toxic polyphenol belonging to the stilbenoid family, naturally found in grapes and berries. Compared to the better-known stilbenoid resveratrol (3,4′,5-trihydroxystilbene), Pt has a dimethoxy group that allows it to be more bioavailable, metabolically stable and, therefore, highly suitable for *in vivo* administration (Kapetanovic et al., 2011; Azzolini et al., 2014). Stilbenes exert several pharmacological activities, including antiproliferative, anti-inflammatory, antiaging, antidiabetic, neuroprotective, and cardioprotective properties (Roupe et al., 2006; Kosuru et al., 2016). The beneficial effects of these compounds lie in their antioxidant properties and in their ability to directly or indirectly modulate signaling pathways also involved in autophagy regulation (Akinwumi et al., 2018).

So far, the proautophagic activity of Pt was mostly investigated in cancer cell lines and tumorigenic conditions (Lee et al., 2019; Ma et al., 2019), whereas much less is known about it in other physiological and pathological conditions. For skeletal muscle, the studied effects of Pt administration have mainly regarded insulin sensitivity (Gómez-Zorita et al., 2015; Tastekin et al., 2018) and muscle adaptation to exercise (Zheng et al., 2020), but autophagy was not investigated in those studies. In light of this, we investigated the autophagy-modulating action of Pt in fibroblast cultures and in skeletal muscle of wild-type and COL6 null mice, and found that Pt treatment potently induces the autophagic flux and is able to ameliorate the muscle pathology of COL6 null animals. These data point at Pt as an effective non-toxic, caloric restriction mimetic that can be exploited for the treatment of autophagy-deficient pathologies.

## Method

### Animals

Six-month-old wild-type and *Col6a1^–/–^* mice in the C57BL/6N background (Irwin et al., 2003) were used. All mice were housed in controlled temperature (23°C) and light (12 h light/12 h dark cycle) conditions, with *ad libitum* access to water and food. Animal procedures were performed according to the Italian laws and approved by the Animal Ethics Committee of the University of Padova (OPBA) and by the Italian Ministry of Health (license protocol n. 480/2019-PR).

### Mouse Treatments

Pt (gently provided by Dr. Mario Zoratti) was dissolved at 500 mM in dimethyl sulfoxide (DMSO, Sigma-Aldrich). Pt stock solution was diluted in distilled water to 90.2 mg/kg body weight in a final volume of 100 μL. Pt was administered by oral gavage for 1 or 5 consecutive days. Control mice received a vehicle solution of DMSO in distilled water. Autophagic flux was investigated by co-treatment with colchicine (Sigma-Aldrich) (Klionsky et al., 2016). Colchicine was dissolved in physiological solution and i.p. injected at 0.4 mg/kg body weight, once a day for 2 consecutive days (Ju et al., 2010). The day after the last injection, mice were sacrificed by cervical dislocation. Once dissected, muscles were quickly frozen in liquid nitrogen-precooled isopentane and stored at −80°C for subsequent histological and biochemical analyses.

### Cell Cultures and Treatments

Primary dermal fibroblasts were isolated from C57BL/6N wild-type mice as described (Takashima, 2001). Briefly, mice were shaved and back skin was removed, washed in phosphate-buffered saline (PBS) supplemented with 3% penicillin-streptomycin (P/S, Life Technologies), and digested for 30 min in 0.25% trypsin-EDTA solution (Thermo Fisher Scientific) at 37°C. Subsequently, the tissue explant was cut into small pieces and placed in a petri dish. The specimens were incubated in Dulbecco’s Modified Eagle’s Medium (DMEM, Thermo Fisher Scientific) supplemented with 20% fetal bovine serum (Thermo Fisher Scientific) and 1% P/S, and maintained at 37°C in 5% CO_2_ until fibroblasts reached confluence. Only P4 to P5 passages from initial fibroblast isolation were used for the experiments. Fibroblasts were treated in the Petri dish for 2.5 h with 15 μM Pt or 0.1% DMSO, added to the culture medium. In order to study the autophagic flux (Klionsky et al., 2016), fibroblasts were co-treated with 50 μM chloroquine (Sigma-Aldrich) during the last 2 h of Pt treatment.

### Western Blotting

Frozen tibialis anterior (TA) muscle was pulverized by grinding in liquid nitrogen and lysed in SDS extraction buffer (50 mM Tris-HCl, pH 7.5; 150 mM NaCl; 10 mM MgCl_2_; 1 mM EDTA; 10% glycerol; 0.5 mM DTT; 2% SDS; 1% Triton X-100) supplemented with protease (Roche) and phosphatase (Sigma-Aldrich) inhibitors. Primary dermal fibroblasts (0.7 × 10^6^ cells) were cultured in 6-well plates. Cells were washed twice with PBS and scraped in NP-40 lysis buffer (50 mM Tris-HCl, pH 7.5; 20 mM EDTA; 150 mM NaCl; 0.5% NP-40) supplemented with protease and phosphatase inhibitors. SDS-PAGE of protein lysates (10–30μg) was carried out in 12% or 4–12% gradient polyacrylamide Novex NuPAGE Bis-Tris gels (Invitrogen), according to protein molecular weight, and electrotransferred onto PVDF membrane (Millipore). Membranes were saturated for 1 h in 5% non-fat milk in Tris-buffered saline containing 0.1% Tween 20 (TBS-T) and incubated overnight at 4°C with the following primary antibodies diluted in 2.5% milk in TBS-T: rabbit anti-LC3B (1:1,000; Thermo Fisher Scientific, PA1-16930); rat anti-LAMP1 (1:300; DSHB, clone 1D4B); guinea pig anti-p62/SQSTM1 (1:300; Santa Cruz Biotechnology, sc-25575); mouse anti-BNIP3 (1:500; Sigma-Aldrich, B7931); mouse anti-GAPDH (1:125,000; Millipore, MAB374); mouse anti-vinculin (1:1,000; Sigma-Aldrich, clone VIN-11-5); mouse anti-β-actin (1:1,500; Sigma-Aldrich, A5316). Horseradish peroxidase-conjugated secondary antibodies (1:2,000; Bethyl Laboratories) were used in 2.5% milk in TBS-T. Signal was detected by chemioluminescence using SuperSignal West Pico (Thermo Fisher Scientific). Densitometric quantification was carried out by ImageJ software (Schneider et al., 2012). When needed, membranes were stripped with an acidic stripping solution (25 mM glycine, 1% SDS, pH 2), saturated and reprobed.

### RNA Extraction and Gene Expression Analyses

Whole frozen tibialis anterior muscles were grinded in liquid nitrogen using pestle and mortar. Total RNA was isolated using TRIzol reagent (Invitrogen), according to manufacturer’s instructions, quantified using Nanodrop ND2000 (Thermo Fisher Scientific) and finally retrotranscribed with the SuperScript III Reverse Transcriptase kit (Thermo Fisher Scientific) using random hexamers. Quantitative PCR was performed on a Rotor-Gene Q Thermal cycler instrument (Qiagen) using SYBR green-containing mastermix (Qiagen). Primer sequences are detailed in Supplementary Table S1. *Actb* was used as a housekeeping gene.

### Muscle Histology

Cryosections of TA muscle (10-μm-thick) were stained with hematoxylin-eosin (Sigma-Aldrich, 51275 and HT110116) following standard protocols. For picrosirius red staining, TA cross-sections were fixed for 15 min in 4% PFA, washed in water and stained for 90 min with Picrosirius Red Stain Kit (Polysciences). Bright-field images were captured using a Leica DM-R microscope equipped with a digital camera. Wheat germ agglutinin (WGA) conjugated with Alexa Fluor 488 (Invitrogen) was used to stain sarcolemma and the extracellular space. TA cross-sections (10-μm-thick) were incubated with WGA (1 μg/mL) and Hoechst 33258 (2.5 μg/mL, Sigma-Aldrich) in PBS for 20 min at room temperature. Slides were washed twice in PBS and mounted in 80% glycerol. Partially overlapping images of the entire muscle section were captured using a Leica DM5000B microscope equipped with a digital camera and stitched with ImageJ software. MATLAB application SMASH (Smith and Barton, 2014) was used to segment the fluorescent stitched images and to measure the cross-sectional area of each myofiber. SMASH parameters were chosen to optimize the segmentation procedure and maintained constant for all the analyses. Wrong selections were manually corrected. Centrally nucleated myofibers, embryonic myosin heavy chain (eMHC)-positive myofibers and the total amount of myofibers per TA section were counted manually.

### Immunofluorescence

Dermal fibroblasts (0.1 × 10^6^ cells) were plated on glass coverslips pre-coated with 0.1% gelatin (Sigma-Aldrich) in PBS. At the end of the treatments described above, cells were washed twice with PBS and incubated in cold 1:1 methanol-acetone solution for 10 min at −20°C. The same fixation/permeabilization procedure was performed on 10-μm-thick cross cryosections of TA muscles. Slides were saturated with 10% goat serum in PBS for 30 min at room temperature and incubated overnight with the following antibodies: rat anti-LAMP1 (1:100; DSHB, clone 1D4B); rabbit anti-LC3B (1:150; Thermo Fisher Scientific, PA1-16930); guinea pig anti-p62/SQSTM1 (1:100; Progen, GP62-C); mouse anti-eMHC (1:25; DSHB, clone F1.652); rabbit anti-Laminin (1:800; Sigma-Aldrich, clone L9393). Slides were washed in PBS and incubated for 1 h at room temperature with the appropriate secondary antibodies (Jackson ImmunoResearch). Slides were mounted in 80% glycerol-PBS and images were taken by using a Zeiss LSM700 laser-scanning confocal microscope.

### *In situ* TUNEL Assay

Quantitative determination of apoptotic cells was performed by terminal deoxynucleotidyl transferase dUTP-mediated nick-end labeling (TUNEL) assay. TA cross-sections (10-μm-thick) were air-dried and apoptotic nuclei were detected by the *In situ* Cell Death Detection Kit TMR red (Roche), according to the manufacture’s guidelines. All nuclei were subsequently counterstained with Hoechst 33258 (Sigma). Random fields were selected using a Leica DM5000B microscope equipped with a digital camera and TUNEL-positive nuclei were manually counted.

### Transmission Electron Microscopy

TA muscles were longitudinally stretched and fixed overnight at 4°C with 2.5% glutaraldehyde (Sigma-Aldrich) and 2% formaldehyde (Sigma-Aldrich) in 0.1 M sodium cacodylate buffer pH 7.4, and postfixed for 2 h at 4°C with 1% osmium tetroxide in 0.1 M sodium cacodylate buffer. After three water washes, samples were dehydrated in a graded ethanol series and embedded in an epoxy resin (Sigma-Aldrich). Ultrathin sections (60–70 nm) were obtained with an Ultrotome V (LKB) ultramicrotome, counterstained with uranyl acetate and lead citrate. Images were acquired with a FEI Tecnai 12 transmission electron microscope (FEI Company) operating at 100 kV and equipped with a Veleta CCD digital camera (Olympus Soft Imaging System).

### Statistics

Comparisons were made by using a two-tailed unpaired Student’s *t*-test and *P* < 0.05 was considered as statistically significant. Data are represented as mean ± s.e.m. The number of biological replicates (always greater than 3) is indicated in each figure caption.

## Results

### Pterostilbene Treatment Elicits Autophagy in Primary Dermal Fibroblasts and Muscles

**FIGURE 1:**
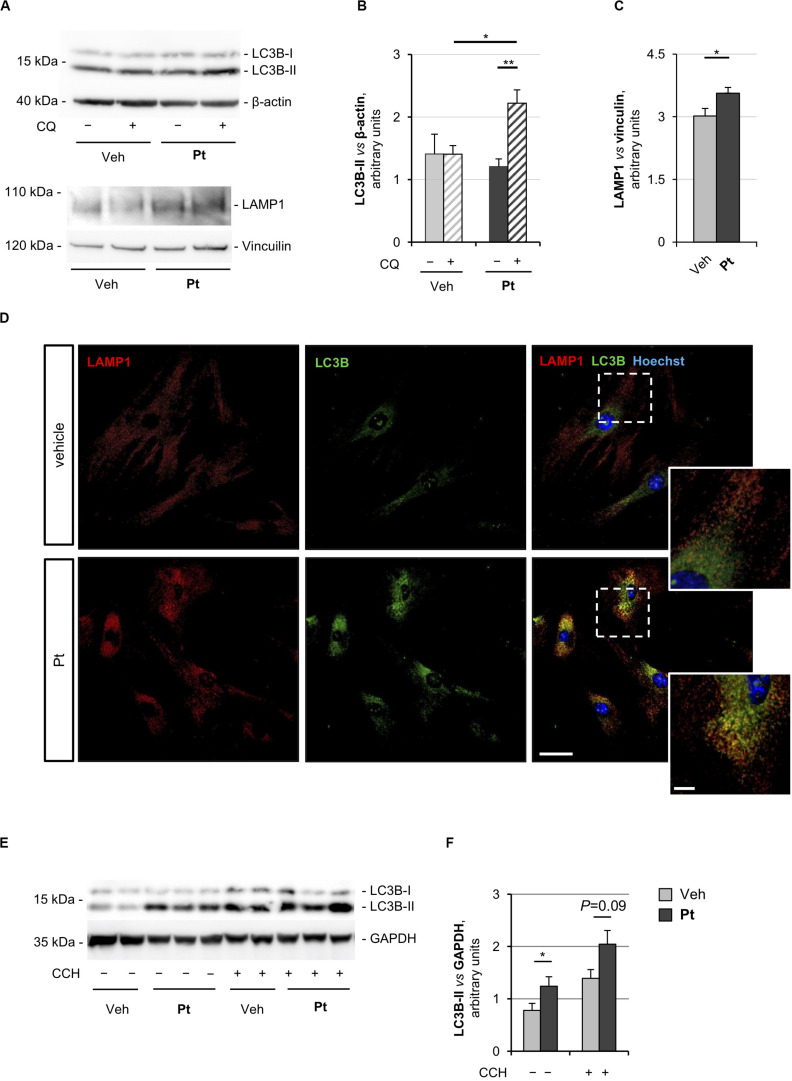
Pterostilbene treatment elicits autophagy in primary dermal fibroblasts and in skeletal muscle of wild-type mice. **(A)** Western blot analysis for LC3B and LAMP1 in protein extracts of primary dermal fibroblasts from wild-type mice, treated with vehicle or with 15 μM Pt and incubated (+) or not (–) with chloroquine (CQ). β-actin and vinculin were used as loading controls. **(B,C)** Densitometric quantifications of LC3B-II vs. β-actin **(B)** and LAMP1 vs. vinculin **(C)**, as determined by at least three independent western blot experiments as in **(A)**. Data are shown as mean ± s.e.m. (*n* = 4–6, each condition; *P < 0.05; **P < 0.01). **(D)** Immunofluorescence confocal images for LAMP1 (red) and LC3B (green) on primary dermal fibroblast cultures from wild-type mice, treated for 2.5 h with 15 μM Pt or with vehicle. Nuclei were counterstained with Hoechst (blue). The dotted area is shown at higher magnifications on the right. Scale bar, 100 or 10 μm (magnifications). **(E)** Western blot analysis for LC3B in protein extracts of TA muscles from wild-type mice treated with a single oral gavage of vehicle or Pt (90.2 mg/kg body weight) and sacrificed 8 h after the treatment. Autophagic flux was assessed by i.p. injection of colchicine (CCH, +) or physiological solution (–). GAPDH was used as a loading control. **(F)** Densitometric quantification of LC3B-II vs. GAPDH, as determined by at least three independent western blot experiments as in **(E)**. Data are shown as mean ± s.e.m. (*n* = 3–4, each condition; *P < 0.05). Veh, vehicle.

The proautophagic properties of Pt had been extensively investigated in immortalized cell lines, but little is known about primary cell cultures. For this reason, we first evaluated the capability of Pt to positively modulate autophagy in primary dermal fibroblasts prepared from wild-type mice. To monitor the autophagic flux, we incubated cells with chloroquine, an inhibitor of lysosome acidification and function (Klionsky et al., 2016). Western blotting showed a significantly higher chloroquine-dependent accumulation of LC3B-II, the best-characterized autophagosome marker, in cultures treated for 2.5 h with Pt when compared to vehicle-treated cultures (Figures 1A,B), indicating that Pt promotes autophagosome formation in primary fibroblasts.

Autophagosome-lysosome fusion represents the final step of the autophagic flux and is equally important for the entire process. Indeed, lysosomes play an essential role in cargo degradation, both in basal condition and during autophagy induction (Huynh et al., 2007; Zhou et al., 2013). In agreement with the Pt-dependent induction of the autophagic flux, primary fibroblasts displayed significantly increased levels of lysosome-associated membrane protein 1 (LAMP1), a *bona fide* marker of the endo-lysosomal compartment, upon Pt treatment (Figures 1A,C). This result was confirmed by immunostaining analysis, showing increased LAMP1-positive signal in fibroblasts upon Pt treatment, when compared to vehicle-treated cells (Figure 1D). When autophagy is activated, the co-localization of autophagosomes with lysosomes reflects the trafficking and degradation of autophagic cargoes. As expected, Pt-treated fibroblasts showed a distinctive increase in the number of LC3B-positive puncta, with extensive co-localization with LAMP1, especially in the perinuclear regions (Figure 1D). Taken together, these data show that Pt is able to induce autophagic flux in primary fibroblast cultures.

In order to evaluate the ability of Pt to induce autophagy *in vivo*, we treated wild-type mice with a single administration of Pt or vehicle via oral gavage and collected skeletal muscles 8 h later. Autophagic flux was assessed by i.p. injection of colchicine, a commonly used alkaloid able to prevent microtubule polymerization (Klionsky et al., 2016). Western blot analysis of protein extracts from TA muscle revealed a significantly higher LC3B lipidation in Pt-treated mice already in basal conditions, without colchicine injection. In addition, enhanced colchicine-dependent LC3B-II accumulation was detectable in Pt-treated mice when compared with vehicle-treated animals (Figures 1E,F). Altogether these results indicate that Pt promotes the autophagic flux in skeletal muscle.

### Pterostilbene Reactivates the Autophagic Flux of Col6a1^–/–^ Myopathic Mice

Considering that defective regulation of autophagy plays a key role in the muscle pathology of both *Col6a1^–/–^* mice and patients affected by COL6-related myopathies (Grumati et al., 2010; Castagnaro et al., 2016), and that its reactivation leads to beneficial effects (Chrisam et al., 2015; Castagnaro et al., 2016), we investigated the effects elicited by Pt administration to *Col6a1^–/–^* mice. Western blot analysis for LC3B lipidation showed that a single Pt treatment by oral gavage was able to elicit a robust increase in LC3B-II protein content in TA muscle of COL6-deficient mice (Supplementary Figure S1). Based on these data, we lengthened the treatment time, in order to allow for muscle remodeling and unravel any phenotypic amelioration.

**FIGURE 2:**
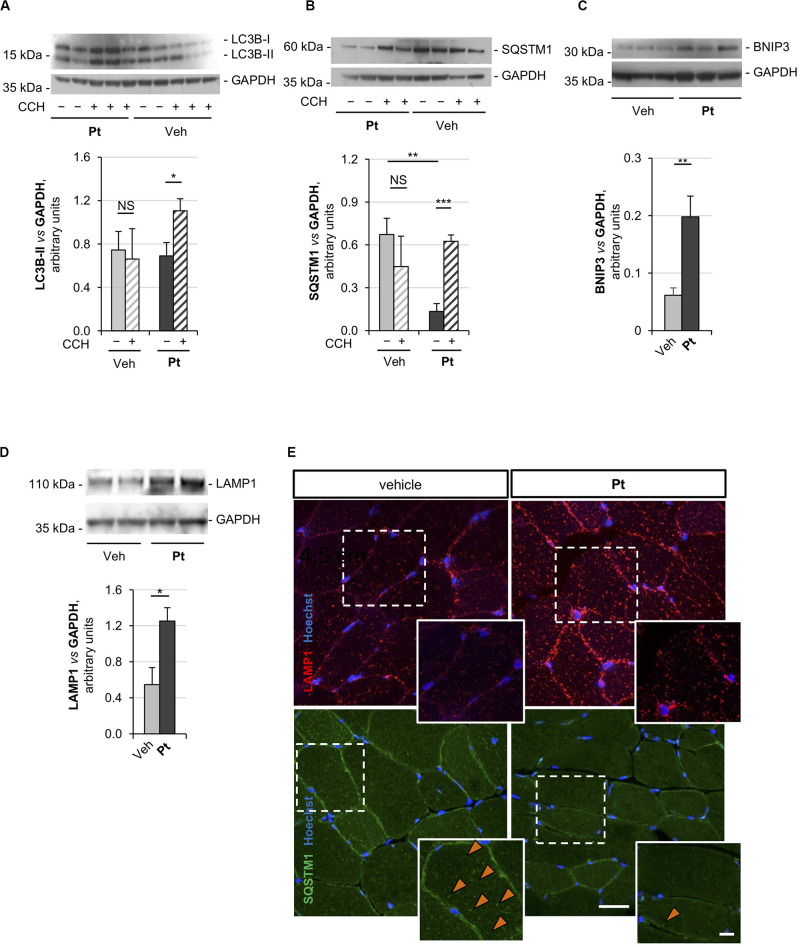
Pterostilbene reactivates the autophagic flux in the skeletal muscle of *Col6a1^–/–^* mice. **(A–D)** Western blot analysis for LC3B **(A)**, p62/SQSTM1 **(B)**, BNIP3 **(C)**, and LAMP1 **(D)** in protein extracts of TA muscles from *Col6a1^–/–^* mice treated by oral gavage for 5 days with vehicle or with Pt (90.2 mg/kg body weight). Autophagic flux was determined by i.p. injection of colchicine (CCH, +) or physiological solution (–). GAPDH was used as a loading control. Densitometric quantifications, as determined by at least three independent western blot experiments, are shown in the respective bottom panels. Data are shown as mean ± s.e.m. (*n* = 4–5, each condition; NS = not significant; *P < 0.05; **P < 0.01; ***P < 0.001). **(E)** Immunofluorescence confocal images for LAMP1 (red) and p62/SQSTM1 (green) in TA cross sections from *Col6a1^–/–^* mice treated by oral gavage for 5 days with vehicle or with Pt (90.2 mg/kg body weight). Nuclei were counterstained with Hoechst (blue). The dotted area is shown at higher magnifications in the smaller panels. Orange arrowheads indicate p62/SQSTM1-positive puncta. Scale bar, 30 or 10 μm (magnifications). Veh, vehicle.

Autophagic flux analysis on skeletal muscle of *Col6a1^–/–^* mice treated with Pt by oral gavage for 5 days revealed strong upregulation of autophagy. Indeed, western blotting on protein extracts from TA muscles showed a significantly higher colchicine-dependent accumulation of LC3B-II in *Col6a1^–/–^* mice treated with Pt when compared to vehicle-treated *Col6a1^–/–^* animals (Figure 2A). As expected, vehicle-treated *Col6a1^–/–^* mice did not display colchicine-dependent LC3B-II accumulation (Figure 2A), in agreement with the previously described autophagy impairment (Grumati et al., 2010). Together with increased LC3B lipidation, Pt-treated *Col6a1^–/–^* mice showed a significant decrease in p62/SQSTM1 protein levels when compared to vehicle-treated animals (Figure 2B), coherently with the higher degradation rate of this autophagic receptor. Conversely, following autophagy flux blockade with colchicine, a significant accumulation of p62/SQSTM1 protein was detectable only in samples from mice treated with Pt (Figure 2B), consistently with the upregulation of autophagosome formation revealed by LC3B-II lipidation.

Previous studies demonstrated that *Col6a1^–/–^* muscles display a down-regulation of the mitophagy mediator BNIP3 (Grumati et al., 2010). Western blot analysis of TA muscle showed a significant increase in BNIP3 protein level in Pt-treated mice compared to the vehicle-treated controls (Figure 2C), suggesting that Pt may be able to promote also mitophagy in *Col6a1^–/–^* muscles. It has been recently demonstrated that the lysosomal compartment is strongly affected in cells lacking COL6 (Castagnaro et al., 2018). TA muscles from Pt-treated *Col6a1^–/–^* mice showed a significant increase in LAMP1 protein levels when compared to vehicle-treated animals (Figure 2D). These findings are in agreement with autophagy upregulation, as an increase in lysosomal content leads to a rapid and efficient autophagosome degradation (Zhou et al., 2013).

The above findings were also confirmed by immunofluorescence microscopy on TA cross-sections, showing a reduction of p62/SQSTM1 puncta and an increase of LAMP1-positive signal in TA sections from Pt-treated *Col6a1^–/–^* mice when compared to vehicle-treated controls (Figure 2E). Altogether, these data indicate that *in vivo* Pt administration leads to an increased rate of autophagosome formation and degradation in the skeletal muscle of autophagy-deficient *Col6a1^–/–^* mice.

### Pterostilbene Remodels COL6-Deficient Skeletal Muscle

**FIGURE 3:**
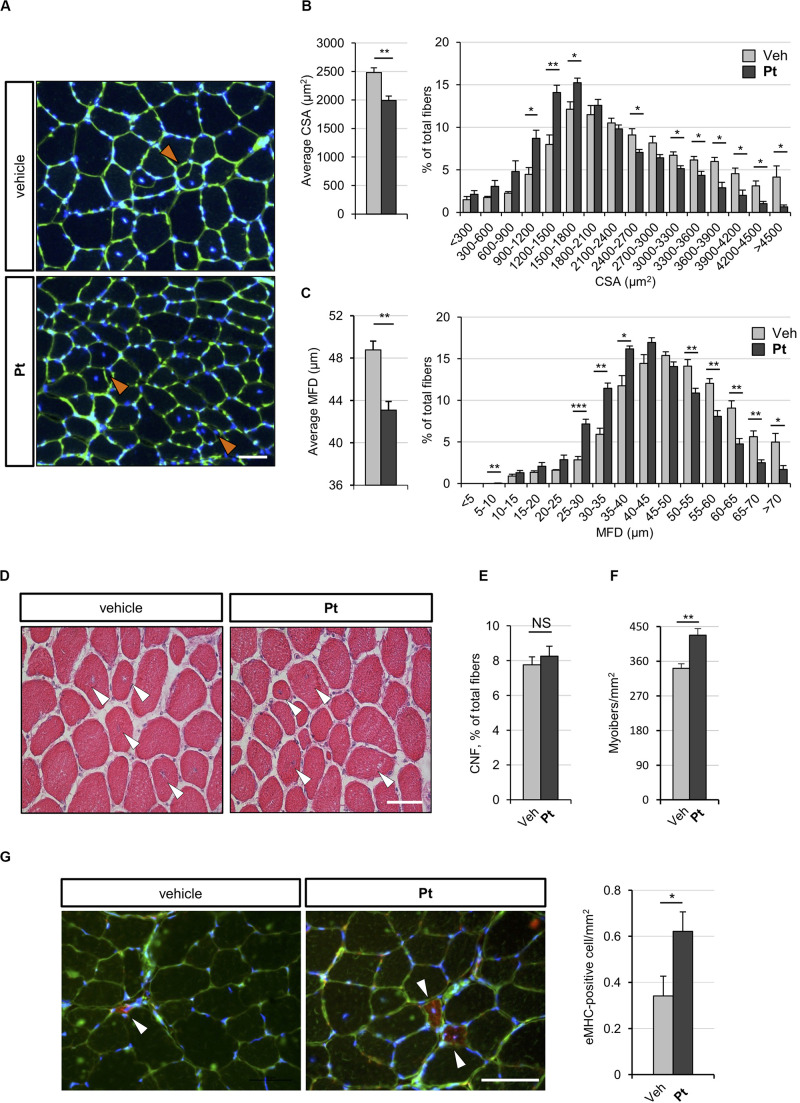
Pterostilbene remodels COL6-deficient skeletal muscle. **(A)** Representative fluorescence microscopy images of TA cross sections of *Col6a1^–/–^* mice treated by oral gavage for 5 days with vehicle or with Pt (90.2 mg/kg body weight) and stained with fluorophore-conjugated wheat germ agglutinin (WGA, green) and Hoechst (blue). Orange arrowheads indicate tiny fibers. Scale bar, 50 μm. **(B)** Morphometric analysis for average cross-sectional area (left panel) and cross-sectional area distribution among myofibers (right panel), as determined from reconstructed images taken from entire TA muscle cross sections as in **(A)**. Data are shown as mean ± s.e.m. (*n* = 4 mice, each condition; *P < 0.05; **P < 0.01). **(C)** Morphometric analysis for average minimum Feret diameter (left panel) and minimum Feret diameter distribution among myofibers (right panel), as determined from reconstructed images taken from entire TA muscle cross sections as in **(A)**. Data are shown as mean ± s.e.m. (*n* = 4 mice, each condition; *P < 0.05; **P < 0.01; ***P < 0.001). **(D)** Hematoxylin-eosin staining of TA cross sections from *Col6a1^–/–^* mice treated by oral gavage for 5 days with vehicle or with Pt (90.2 mg/kg body weight). White arrowheads point at centrally located myonuclei. Scale bar, 50 μm. **(E,F)** Average percentage of centrally nucleated myofibers per muscle section **(E)** and average number of myofibers per unit area **(F)**, as determined on reconstructed images taken from whole TA cross sections as in **(D)**. Data are shown as mean ± s.e.m. (*n* = 4; NS = not significant;**P < 0.01). **(G)** Representative fluorescence microscopy images of TA cross sections of *Col6a1^–/–^* mice treated by oral gavage for 5 days with vehicle or with Pt (90.2 mg/kg body weight) and stained with antibodies for eMHC (red) and laminin (green). Nuclei were counterstained with Hoechst (blue). White arrowheads indicate eMHC-positive fibers. On the right, quantification of eMHC-positive cells per area unit (in mm^2^). Data are shown as mean ± s.e.m. (*n* = 6; *, *P* < 0.05). Scale bar, 50 μm. CSA, cross-sectional area; MFD, minimum ferret diameter; Veh, vehicle.

To assess whether the autophagy induction promoted by Pt administration is accompanied by skeletal muscle remodeling, we carried out histological and morphometric analyses on muscles from Pt- and vehicle-treated *Col6a1^–/–^* mice. Five days of Pt treatment led to a significant decrease in the average cross-sectional area and minimum Feret diameter of *Col6a1^–/–^* myofibers (Figures 3A–C). Accordingly, by plotting the distribution of cross-sectional area and minimum Feret diameter among the analyzed myofibers, a shift toward smaller fiber classes was evident in Pt-treated mice (Figures 3B,C). These findings support the concept of catabolic effects elicited by Pt in *Col6a1^–/–^* muscles, in line with robust autophagy induction. On the other side, Pt does not activate an atrophy process mediated by atrogenes, as highlighted by transcript analysis for Murf-1*/Trim63* and Atrogin-1*/Fbxo32* (Supplementary Figure S2A). Hematoxylin-eosin staining on TA cross-sections did not reveal any detrimental changes in muscle architecture of Pt-treated mice (Figure 3D), and Sirius red staining confirmed that there was no noticeable sign of fibrosis in Pt-treated mice (Supplementary Figure S2B). Furthermore, the percentage of centrally nucleated fibers was similar between Pt- and vehicle-treated animals (Figure 3E), and was consistent with previously published data for COL6-deficient muscles (Bonaldo et al., 1998). Interestingly, upon 5 days of Pt treatment *Col6a1^–/–^* muscles displayed a significantly higher number of myofibers per area unit (Figure 3F) and this was accompanied by a significant increase of newly formed, eMHC-positive myofibers (Figure 3G).

Altogether these results support the concept that Pt treatment promotes myofiber remodeling due to both increased autophagy and enhanced regeneration.

### Pterostilbene Treatment Ameliorates the Myopathic Phenotype of Col6a1^–/–^ Mice

**FIGURE 4:**
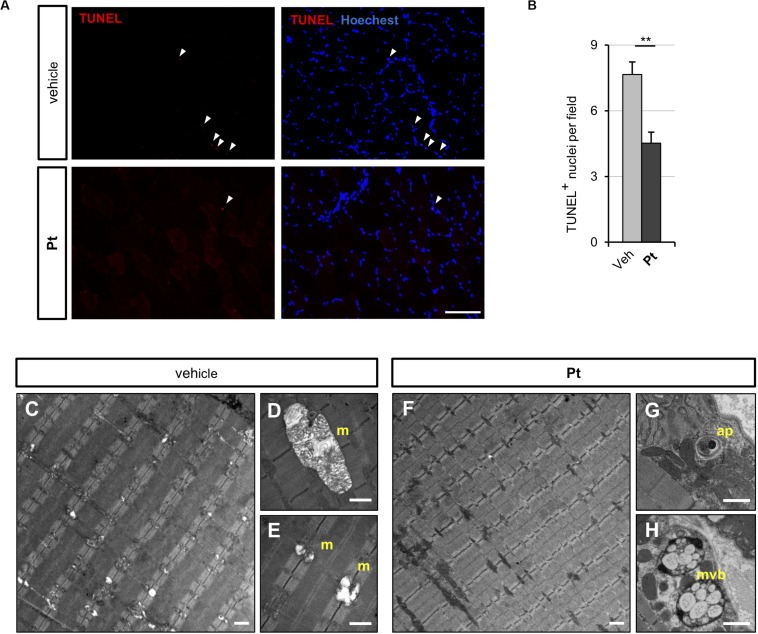
Pterostilbene treatment ameliorates the myopathic phenotype of Col6a1^–/–^ mice. **(A)** Representative fluorescence microscopy images of TA cross sections of Col6a1^–/–^ mice treated for 5 days with vehicle or with Pt (90.2 mg/kg body weight) and stained for apoptotic nuclei (TUNEL assay, red) and Hoechst (blue). White arrowheads indicate TUNEL-positive nuclei. Scale bar, 100 μm. **(B)** Quantification of TUNEL-positive myonuclei in TA cross sections from Col6a1^–/–^ mice treated with by oral gavage for 5 days with vehicle or with Pt (90.2 mg/kg body weight). Data are shown as mean ± s.e.m. (n = 4; **P < 0.01). **(C–H)** Transmission electron microscopy images of TA longitudinal sections from Col6a1^–/–^ mice treated by oral gavage for 5 days with vehicle **(C–E)** or with Pt (90.2 mg/kg body weight) **(F–H)**. Scale bar, 1 μm. ap, autophagosome; m, mitochondrion; mvb, multivesicular body; Veh, vehicle.

Since the above data showed that Pt promotes autophagy *in vivo* and rescues the autophagy impairment of COL6-deficient skeletal muscle, we evaluated whether Pt treatment is able to ameliorate the myopathic phenotype of *Col6a1^–/–^* mice. Spontaneous myofiber apoptosis is a typical feature of COL6-related myopathies and its amelioration is beneficial both in *Col6a1^–/–^* murine model and in BM and UCMD patients (Chrisam et al., 2015; Castagnaro et al., 2016). TUNEL assay revealed a significant decrease in the incidence of apoptotic myonuclei in TA of *Col6a1^–/–^* mice treated for 5 days with Pt (Figures 4A,B), thus showing that Pt is able to counteract myofiber apoptosis in COL6-deficient muscle.

Abnormal mitochondria with altered cristae and dilated sarcoplasmic reticulum are distinctive ultrastructural defects of COL6-deficient muscles (Irwin et al., 2003; Grumati et al., 2010). Transmission electron microscopy of TA cross-sections showed that 5 days of Pt treatment led to a strong amelioration of muscle ultrastructure, with a dramatic decrease of mitochondrial swelling and dilated sarcoplasmic reticulum in Pt-treated *Col6a1^–/–^* mice when compared to vehicle-treated animals (Figures 4C–H). Moreover, the muscles of Pt-treated animals displayed autophagic vacuoles and multivesicular bodies (Figures 4G,H), supporting autophagy induction and recycling of damaged organelles triggered by Pt administration (Fader and Colombo, 2009). Five-day Pt treatment did not lead to any overt alteration in sarcomeric and sarcolemmal ultrastructure (Figure 4G and data not shown).

Altogether these data confirm the beneficial effect of Pt in counteracting the pathogenic hallmarks of *Col6a1^–/–^* muscles.

## Discussion

Autophagy deregulation is linked to the pathogenesis of several disorders, including muscle congenital diseases. In *Col6a1^–/–^* mice, the best-characterized animal model of COL6-related myopathies, defective regulation of the autophagic process leads to the accumulation of dysfunctional and harmful organelles, which dramatically affects myofiber viability (Grumati et al., 2010). This is exacerbated by impaired muscle regeneration, as COL6 is a key component of the muscle stem cell niche and its absence compromises the self-renewal capability of satellite cells upon muscle injury (Urciuolo et al., 2013). Two pharmacological approaches have so far been assessed in the preclinical setting, based on rapamycin and cyclosporin A treatments (Grumati et al., 2010; Urciuolo et al., 2013; Gattazzo et al., 2014). The proautophagic and promyogenic properties of these two drugs are well known, but their strong immunosuppressive activity makes them unsuitable for the long-term chronic treatment of patients affected by muscular dystrophies (Merlini et al., 2011). To overcome this unwanted side effect, a drug-free approach, based on a low protein diet, has been tested both in the preclinical setting and in a pilot clinical trial, confirming its beneficial effect in terms of muscle structure and function (Grumati et al., 2010; Castagnaro et al., 2016). However, this dietary regimen requires quite a profound change in food habits and it is not recommended for infants or most severely affected patients.

A promising way to rescue the myopathic phenotype of COL6 deficient mice consists in the administration of nutraceutical autophagy-inducing compounds. Nutraceuticals represent non-toxic molecules, generally found in food, able to provide health benefits and exploitable as a dietary supplement. The polyamine spermidine and the polyphenol resveratrol are among the most characterized ones. Their ability to boost autophagy is well known, as they act as caloric restriction mimetics through different but converging molecular pathways (Morselli et al., 2011). Spermidine was already demonstrated to display beneficial effects in the myopathic *Col6a1^–/–^* mouse model (Chrisam et al., 2015), however nothing is known about the therapeutic potential of resveratrol in this myopathic condition.

The major aim of the present study was to investigate the ability of Pt, a resveratrol analog characterized by higher bioavailability and chemical stability (Kapetanovic et al., 2011), to promote autophagy in skeletal muscle. Indeed, this stilbenoid was previously shown to have antioxidant and pro-autophagic properties in various pathophysiological conditions (Akinwumi et al., 2018), however no study has ever exploited its effects on autophagy in skeletal muscle. Our data in wild-type mice indicate that Pt exerts a strong promoting effect on the autophagic flux of skeletal muscle already after 8 h from oral administration, suggesting that it has a rapid onset of action, according to the post-translational nature of phagophore nucleation. Based on these data, we moved forward performing a 5-day-long Pt treatment in COL6 null mice, in which autophagy activation in skeletal muscle is resistant even to 24-h fasting (Grumati et al., 2010). Pt strongly reactivates the processes of autophagosome formation and degradation in *Col6a1^–/–^* mice under fed condition, together with an increase in lysosomal content, thus highlighting the high pro-autophagic efficacy of this stilbenoid molecule. These effects, together with the Pt-induced myofiber regeneration, result in the observed skeletal muscle remodeling in COL6 null mice, manifesting as a decreased myofiber size and an increased number of newly forming myofibers. This suggests a prospective translational relevance for COL6 pathologies, as stimulating myogenesis in COL6-deficient muscles is beneficial both in the *Col6a1^–/–^* animal model and in UCMD patients (Merlini et al., 2011; Gattazzo et al., 2014).

The high incidence of apoptotic myofibers is a well-characterized feature of COL6-related myopathies (Irwin et al., 2003; Angelin et al., 2007; Merlini and Bernardi, 2008; Grumati et al., 2010). Pt has a remarkable effect in decreasing the incidence of TUNEL-positive nuclei in *Col6a1^–/–^* muscle, pointing at increased myofiber viability in COL6-deficient muscles following Pt administration. This anti-apoptotic effect of Pt is likely due to the autophagy-mediated clearance of swollen mitochondria and dilated sarcoplasmic reticulum, since a marked recovery of organelle ultrastructure is clearly visible after 5-day Pt treatment. Indeed, it is well known that a compromised mitochondrial network is prone to release several proapoptotic factors, detrimental for cell survival.

Of note, despite the remarkable proautophagic effects of 5-day Pt treatment in COL6 null mice, muscle ultrastructure was positively affected, suggesting that the Pt-promoted induction of the autophagic flux is not excessive, but instead well-tolerated and with a beneficial outcome in COL6 deficient muscles. Future work will allow ascertaining the lowest, but still effective, Pt dosages able to elicit functional outcomes, as muscle strength recovery, in *Col6a1^–/–^* mice under long-term administration regimens. Furthermore, it will be interesting to exploit the possible synergistic effects of Pt with other drugs or natural compounds, such as non-immunosuppressive cyclosporin A derivatives (Zulian et al., 2014) or spermidine (Chrisam et al., 2015).

Altogether, our findings point at Pt as a promising autophagy-inducing nutraceutical for skeletal muscle, able to counteract the myopathic phenotype of *Col6a1^–/–^* mice. Indeed, our data indicate that Pt is an excellent nutraceutical with a valuable prospective medical relevance for COL6-related myopathies, as well as for other pathological conditions characterized by an autophagy deficiency, such as Duchenne muscular dystrophy and age-related sarcopenia.

## Data Availability Statement

The raw data supporting the conclusions of this article will be made available by the authors, without undue reservation, to any qualified researcher.

## Ethics Statement

The animal study was reviewed and approved by the Animal Ethics Committee of the University of Padova (OPBA); Italian Ministry of Health (license protocol n. 480/2019-PR).

## Author Contributions

SM and LG conceptualized and performed the experiments, analyzed and interpreted the data, and wrote the manuscript. MC performed muscle autophagy analysis. PBr contributed to mouse treatments. MB and BB contributed to muscle experiments. PBo coordinated the study and contributed to manuscript writing and revision. ML contributed to mouse treatments. All authors contributed to the article and approved the submitted version.

## Conflict of Interest

The authors declare that the research was conducted in the absence of any commercial or financial relationships that could be construed as a potential conflict of interest.

## Supplementary Material

FIGURE S1 A single bout of pterostilbene induces LC3B lipidation in *Col6a1^–/–^* mice. Western blot analysis for LC3B (left panel) and relative densitometric quantification (right panel) in protein extracts of TA muscle from *Col6a1^–/–^* mice treated with a single oral gavage of vehicle or Pt (90.2 mg/kg body weight) and sacrificed 8 h after the treatment. GAPDH was used as a loading control. Data are shown as mean ± s.e.m. (*n* = 3–4; ^∗^*P* < 0.05). Veh, vehicle.

FIGURE S2 Pterostilbene remodels COL6-deficient skeletal muscle. **(A)** Quantitative RT-qPCR analysis of mRNA levels for Atrogin1 (*Fbxo32*), MURF-1 (*Trim63*), and Cathepsin L (*Ctsl*) in RNA extracts of TA muscle from *Col6a1^–/–^* mice treated by oral gavage for 5 days with vehicle or with Pt (90.2 mg/kg body weight). Data are shown as mean ± s.e.m. (*n* = 5; ^∗^*P* < 0.05). **(B)** Picrosirius-red staining of TA cross sections from *Col6a1^–/–^* mice treated by oral gavage for 5 days with vehicle or with Pt (90.2 mg/kg body weight). Scale bar, 50 μm. Veh, vehicle.
