# Corrigendum: The Polyphenol Pterostilbene Ameliorates the Myopathic Phenotype of Collagen VI Deficient Mice via Autophagy Induction

**DOI:** 10.3389/fcell.2020.615542

**Published:** 2021-01-07

**Authors:** Samuele Metti, Lisa Gambarotto, Martina Chrisam, Martina La Spina, Martina Baraldo, Paola Braghetta, Bert Blaauw, Paolo Bonaldo

**Affiliations:** ^1^Department of Molecular Medicine, University of Padova, Padua, Italy; ^2^Department of Biomedical Sciences, University of Padova, Padua, Italy; ^3^Venetian Institute of Molecular Medicine, Padua, Italy; ^4^CRIBI Biotechnology Center, University of Padova, Padua, Italy

**Keywords:** skeletal muscle, autophagy, muscle remodeling, congenital muscular dystrophies, nutraceutical agent, Collagen VI

“Martina La Spina” was not included as an author in the published article. The corrected Author Contributions Statement appears below.

“SM and LG conceptualized and performed the experiments, analyzed and interpreted the data, and wrote the manuscript. MC performed muscle autophagy analysis. PBr contributed to mouse treatments. MB and BB contributed to muscle experiments. PBo coordinated the study and contributed to manuscript writing and revision. ML contributed to mouse treatments. All authors contributed to the article and approved the submitted version.”

The authors apologize for this error and state that this does not change the scientific conclusions of the article in any way. The original article has been updated.

