# Supplementary material for: The Polyphenol Pterostilbene Ameliorates the Myopathic Phenotype of Collagen VI Deficient Mice via Autophagy Induction
Source: Front Cell Dev Biol. 2020 Sep 29;8:580933. doi: 10.3389/fcell.2020.580933 (PMC7550465; doi:10.3389/fcell.2020.580933)
Supplement: FIGURE S1 — A single bout of pterostilbene induces LC3B lipidation in Col6a1–/– mice. Western blot analysis for LC3B (left panel) and relative densitometric quantification (right panel) in protein extracts of TA muscle from Col6a1–/– mice treated with a single oral gavage of vehicle or Pt (90.2 mg/kg body weight) and sacrificed 8 h after the treatment. GAPDH was used as a loading control. Data are shown as mean ± s.e.m. (n = 3–4; ∗P < 0.05). Veh, vehicle. [file Data_Sheet_1.PDF]

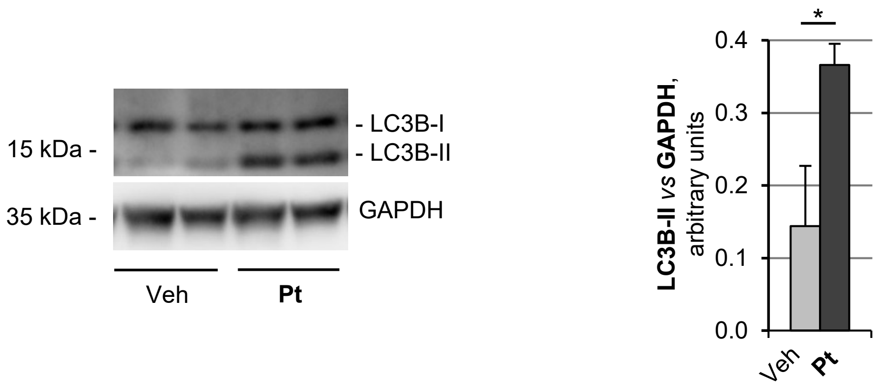


# Supplementary Figure S1

A

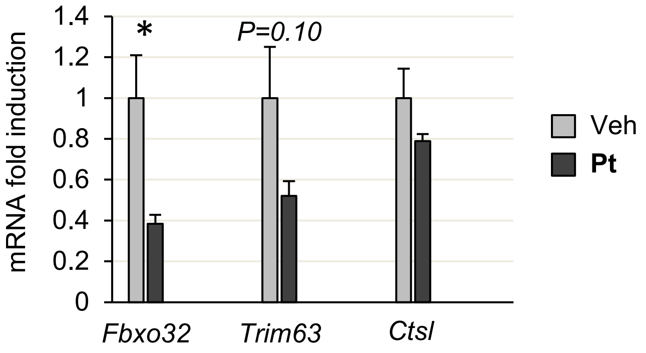


B

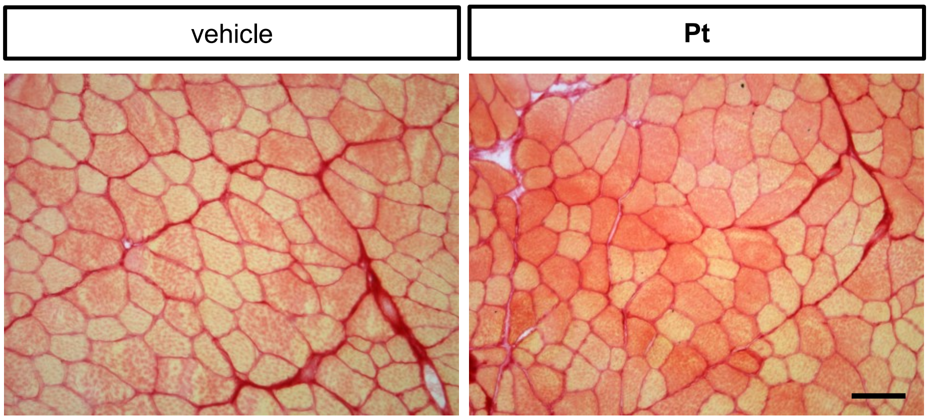


Supplementary Figure S2

| Gene name | Primer sequence (5’ - 3’) | |
|---|---|---|
| *Actb* | Fw: CTAAGGCCAACCGTGAAAAG | Rv: ACCAGAGGCATACAGGGACA |
| *Fbxo32* | Fw: GCAAACACTGCCACATTCTCTC | Rv: CTTGAGGGGAAAGTGAGACG |
| *Murf1* | Fw: ACCTGCTGGTGGAAAACATC | Rv: CTTCGTGTTCCTTGCACATC |
| *Ctsl* | Fw: GTGGACTGTTCTCACGCTCAAG | Rv: TCCGTCCTTCGCTTCATACG |

# Supplementary Table 1
